# Dermographism in chronic spontaneous urticaria: associations with disease activity, autoimmunity, and disease duration

**DOI:** 10.3389/fimmu.2026.1907505

**Published:** 2026-08-03

**Authors:** Vesna Trajkova, Natasa Teovska Mitrevska, Nevenka Velickova, Kanokvalai Kulthanan, Daria Fomina, Philip Hei Li, Hristina Breshkovska, Daniela Buklioska Ilievska

**Affiliations:** 1 Department of Dermatology, General Hospital “8th September”, Skopje, North Macedonia; 2 Higher Medical School, University St. Kliment Ohridski, Bitola, North Macedonia; 3 Dermatology Department, Remedica Hospital, Skopje, North Macedonia; 4 Faculty of Dental Medicine, Dermatology Department, International Balkan University, Skopje, North Macedonia; 5 Faculty of Medical Sciences, Goce Delcev University, Stip, North Macedonia; 6 Department of Dermatology, Faculty of Medicine Siriraj Hospital, Mahidol University, Bangkok, Thailand; 7 Moscow Research and Clinical Center of Allergy and Immunology, City Clinical Hospital 52, Moscow, Healthcare Department, Moscow, Russia; 8 Department of Clinical Immunology and Allergology, I.M. Sechenov First Moscow, State Medical University (Sechenov University), Moscow, Russia; 9 Department of Pulmonology, Astana Medical University, Astana, Kazakhstan; 10 Division of Rheumatology & Clinical Immunology, Department of Medicine, Queen Mary Hospital, The University of Hong Kong, Hong Kong, Hong Kong SAR, China; 11 Department of Dermatology, Faculty of Medicine, Skopje, North Macedonia; 12 Pulmonary Department, General Hospital “8th September”, Skopje, North Macedonia

**Keywords:** associations with disease activity, ASST, autoimmunity, chronic spontaneous urticaria, dermographism

## Abstract

**Background and objective:**

Dermographism is a frequent clinical finding in patients with chronic spontaneous urticaria (CSU) and may reflect enhanced mast-cell reactivity, increased disease severity, and prolonged disease persistence. This study aimed to evaluate the clinical significance of dermographism in CSU, including its relationship with disease activity, disease duration, response to H1-antihistamines, autoimmune status, and autologous serum skin test (ASST) positivity.

**Methods:**

A prospective observational study was conducted at the Dermatology Department, General Hospital “8th September”, Skopje, North Macedonia, between December 2021 and November 2022, including 230 patients with CSU. Disease activity was assessed using the Urticaria Activity Score over 7 days (UAS7). Dermographism was clinically evaluated during dermatological examination using standardized mechanical provocation testing. Autoimmune status was determined by the presence of autoimmune disease and/or positivity for autoantibodies including anti-thyroid peroxidase antibodies (anti-TPO), antinuclear antibodies (ANA), and rheumatoid factor (RF). ASST was performed to assess autoreactivity.

**Results:**

Among 230 patients with chronic spontaneous urticaria (CSU), dermographism was present in 161 (70.0%). Patients with dermographism had significantly higher disease activity, being more frequently classified as severe according to UAS7 compared with those without dermographism (82.0% vs. 18.0%, χ²=14.4, p=0.00015). Dermographism was not associated with therapeutic response to H1-antihistamines (69.3% vs. 30.7%, χ²=0.12, p=0.73), autoimmune status (72.4% vs. 27.6%, χ²=1.1, p=0.29), or ASST positivity (72.9% vs. 27.1%, χ²=1.4, p=0.24). Patients with dermographism had a significantly longer disease duration than those without dermographism (58.5 ± 47.1 months; median 54 [IQR 15–91] months, Z = 2.3, p=0.022).

**Conclusions:**

Dermographism is a common finding in patients with chronic spontaneous urticaria and is associated with greater disease activity and longer disease duration, suggesting a more severe and persistent disease phenotype. Despite the observed trends, no significant associations were identified between dermographism and H1-antihistamine response. In addition, the lack of significant associations with autoimmune status and ASST positivity does not support a definitive relationship between dermographism and immune dysregulation. Routine assessment of dermographism may help identify CSU patients at risk for increased disease burden and prolonged disease course.

## 1 Introduction

Chronic spontaneous urticaria (CSU) is a heterogeneous inflammatory skin disorder characterized by the recurrent spontaneous appearance of wheals, angioedema, or both for a duration exceeding six weeks in the absence of identifiable provoking factors (1, 2). Chronic inducible urticaria (CIndU) is a form of chronic urticaria in which wheals and/or angioedema are consistently triggered by specific stimuli, including cold, heat, pressure, vibration, sunlight, water, exercise, and mechanical skin stroking (symptomatic dermographism) (1, 2). Symptomatic dermographism has an adjusted point prevalence of 3.20% and a lifetime prevalence of 5.94%, with the highest rates in female individuals aged 25–60 years (3, 4). The disease substantially impairs quality of life due to persistent pruritus, visible skin lesions, sleep disturbances, psychological stress, and social impairment (5, 6).

Dermographism, also referred to as “skin writing,” represents an exaggerated wheal-and-flare response induced by mechanical stimulation of the skin. Dermographism comprises a spectrum of mechanically induced cutaneous responses ranging from physiological red dermographism (PD), characterized by transient linear erythema without wheal formation or pruritus, through simple urticarial dermographism (SimUD), defined by scratching-induced wheals in the absence of symptoms, to symptomatic dermographism (SD), in which whealing is accompanied by pruritus or burning sensations; however, there is currently no consensus definition for red dermographism, with ongoing controversy regarding its nosological classification (3, 4, 7–9). Although classically categorized among chronic inducible urticarias, dermographism is frequently observed in patients with CSU and may coexist with spontaneous wheals and pruritus (7, 8).

The underlying pathophysiological mechanisms involve mast-cell and basophil activation with subsequent release of histamine, leukotrienes, cytokines, and additional proinflammatory mediators (1–3, 10). In CSU patients exhibiting dermographism, mechanical stimulation may lower the activation threshold for mast-cell degranulation, thereby amplifying cutaneous inflammatory responses (10–12). Previous studies have suggested that the coexistence of dermographism with CSU may indicate greater disease activity, increased symptom burden, impaired quality of life, and prolonged disease duration (1–3, 6, 7).

Increasing evidence also supports an autoimmune basis in a substantial proportion of CSU patients, particularly involving functional autoantibodies targeting FcϵRIα or IgE (11, 12). Autoimmune mechanisms may contribute not only to spontaneous wheal formation but also to enhanced skin reactivity and inducible symptoms such as dermographism (13). Furthermore, ASST positivity has been associated with autoreactive CSU phenotypes characterized by more severe and persistent disease (14).

Despite the frequent coexistence of dermographism and chronic spontaneous urticaria (CSU), the clinical implications of dermographism in CSU remain incompletely understood. In particular, its relationship with disease severity, chronicity, treatment response, and immunological characteristics has not been fully elucidated. Therefore, the aim of this study was to investigate the prevalence and clinical significance of dermographism in patients with CSU and to determine whether the presence of dermographism is associated with increased disease activity, prolonged disease duration, response to H1-antihistamine therapy, autoimmune status, and autologous serum skin test (ASST) positivity.

## 2 Materials and methods

### 2.1 Patient selection

Adult participants aged 18 years or older with a confirmed diagnosis of active chronic spontaneous urticaria (CSU) were enrolled according to the diagnostic criteria established by the EAACI/GA²LEN/EuroGuiDerm/APAAACI guideline (1). Active CSU was defined by the recurrent spontaneous occurrence of wheals, angioedema, or both for a duration exceeding six consecutive weeks in the absence of identifiable provoking factors. Patients with inducible forms of urticaria, including cold urticaria, cholinergic urticaria, delayed pressure urticaria, solar urticaria, vibratory angioedema, or other physical urticarias except symptomatic dermographism, were excluded from participation. Individuals with bradykinin-mediated angioedema were also excluded.

The present investigation was initially conceived as a comprehensive evaluation of 230 patients with CSU, within which dermographism was analyzed as a relevant clinical parameter associated with disease activity, autoreactivity, and disease persistence. The study was approved by the Ethics Committees of General Hospital 8th September, Skopje (Approval No. 2796), and the Faculty of Medical Sciences, Goce Delcev University, Stip (Approval No. 2005-35/1), and was conducted in accordance with the Declaration of Helsinki.

Although recruitment and clinical assessments were performed at the Dermatology Department of the General Hospital “8th September” in Skopje, the institution functions as a secondary referral center, enabling the inclusion of patients from multiple geographic regions throughout North Macedonia.

The study cohort reflected the ethnic diversity of the country and included ethnic Macedonians, Albanians, Turks, and Roma patients. Participant recruitment was not influenced by ethnicity, and no clinically meaningful differences in CSU manifestations, dermographism frequency, or disease severity were identified among the different ethnic groups. This multiethnic representation enhances the external validity of the findings and supports their applicability to the broader population of CSU patients in the region.

### 2.2 Clinical assessments

At study entry, demographic and clinical variables were systematically documented using standardized case-report forms. Recorded parameters included age, sex, age at disease onset, disease duration story, concomitant autoimmune diseases, and previous or ongoing therapeutic interventions.

Disease activity was evaluated using the Urticaria Activity Score over 7 days (UAS7), which combines daily wheal counts and pruritus intensity recorded over one week. According to validated cutoff values, UAS7 scores were categorized as severe (28–42), moderate (16–27), mild (7–15), well-controlled (1–6), and complete remission (0) (15, 16).

Dermographism was assessed clinically during dermatological examination by applying standardized mechanical pressure to the skin using a blunt instrument. A positive response was defined as the appearance of a linear wheal-and-flare reaction within 10 minutes after provocation (3, 4). Only symptomatic dermographism (SD) was assessed in this study; red dermographism without wheal formation was not recorded. As standardized instruments such as the FricTest or a dermographometer were not used for provocation testing, all examinations were performed by the same physician to minimize inter-operator variability. The presence or absence of dermographism was documented for all participants and subsequently correlated with disease activity, autoimmune markers, treatment responsiveness, ASST reactivity, and disease duration.

Therapeutic response was evaluated during follow-up visits. Patients were classified as responders when disease control was achieved with a UAS7 ≤ 7 while receiving up to fourfold doses of second-generation non-sedating H1-antihistamines. Patients with persistent disease activity despite standard or escalated antihistamine therapy were categorized as non-responders (17).

Disease duration was calculated as the interval between initial symptom onset and study inclusion. Recurrence was defined as the reappearance of wheals and/or dermographic symptoms following a symptom-free interval of at least six months (17).

### 2.3 Assessment of autoimmune status

Autoimmune status was determined based on documented autoimmune disease or serological positivity for at least one autoimmune biomarker, including anti-thyroid peroxidase antibodies (anti-TPO), antinuclear antibodies (ANA), or rheumatoid factor (RF) (17). Patients were subsequently classified as having a positive or negative autoimmune status based on the presence or absence of documented autoimmune disease and/or serological positivity for at least one autoimmune biomarker (17).

### 2.4 Autologous serum skin test

The autologous serum skin test (ASST) was performed as an *in vivo* indicator of autoreactivity. To reduce the possibility of false-negative results, antihistamines were discontinued for a minimum of 48 hours prior to testing, H2-receptor antagonists and leukotriene receptor antagonists for at least 7 days, and systemic corticosteroids or cyclosporine for at least one month before the procedure (14, 17).

Approximately 5 mL of venous blood was obtained from each participant under sterile conditions. Blood samples were left undisturbed at room temperature for 30 minutes to allow clot formation and were subsequently centrifuged at 2,000 × g for 15 minutes to isolate serum (14, 17).

For the ASST procedure, 0.05 mL of autologous serum was injected intradermally into the volar aspect of the forearm. Histamine and physiological saline were simultaneously administered as positive and negative controls, respectively. Reactions were evaluated after 30 minutes. ASST positivity was defined as a serum-induced wheal exceeding the saline control by more than 1.5 mm in mean diameter (14, 17).

### 2.5 Statistical analysis

Statistical analyses were performed using SPSS software version 23 (IBM Corp., Armonk, NY, USA). The distribution of continuous variables was assessed using the Kolmogorov-Smirnov and Shapiro-Wilk normality tests. Parametric variables were analyzed using Student’s t-test for comparisons between two groups and one-way ANOVA for comparisons involving multiple groups. Non-parametric variables were evaluated using the Mann-Whitney U test or Kruskal-Wallis test where appropriate.

Categorical variables were analyzed using the Chi-square test or Fisher’s exact test when expected cell counts were below five. All analyses were two-sided, and p-values below 0.05 were considered statistically significant (18).

## 3 Results

### 3.1 Occurrence of dermographism and disease severity in CSU patients

As shown in **Table 1**, dermographism was present in 161 (70.0%) patients. Dermographism was significantly more frequent among individuals with severe disease activity according to UAS7 categorization. Patients with severe CSU demonstrated a markedly higher prevalence of dermographism compared with patients exhibiting moderate, mild, or well-controlled disease activity, A significant association was observed between dermographism and severe disease activity compared with moderate disease activity (χ²=14.4, p=0.00015).

**Table 1:** Dermographism and disease severity.

Variable	UAS7 Disease activity in CSU					
	n	Severe n (%)	Moderate n (%)	Mild n (%)	Well-controlled n (%)	No symptoms n (%)
Dermographism						
Present	161	73 (82.02)	36 (69.23)	24 (51.06)	14 (63.64)	14 (70)
Not present	69	16 (17.98)	16 (30.77)	23 (48.94)	8 (36.36)	6 (30)
p-level	severe/moderate X^2^ = 14.4 *p=0.00015					

### 3.2 Dermographism and therapeutic respond to H1-antihistamines in CSU patients

As shown in **Table 2**, no significant association was observed between dermographism and therapeutic response to H1-antihistamines in CSU patients, with comparable response rates in patients with and without dermographism (69.3% vs. 71.6%, respectively; χ²=0.12, p=0.73).

**Table 2:** Dermographism and therapeutic response in CSU patients.

Variable	Therapeutic Respond to H1-antihistamines			p-level
	n	Present n (%)	Not present n (%)	
Dermographism				
Present	161	113 (69.33)	48 (71.64)	X^2^ = 0.12 p=0.73
Not present	69	50 (30.67)	19 (28.36)	

### 3.3 Association between dermographism and autoimmune status in CSU patients

Among patients with CSU, 51.3% had a documented autoimmune disease, most commonly Hashimoto thyroiditis (28.7%), followed by vitiligo (8.7%), rheumatoid arthritis (6.09%), Crohn’s disease (3.48%), and concomitant Hashimoto thyroiditis and vitiligo (4.35%); additionally, autoantibody positivity was observed in a substantial proportion of patients, with ANA positivity in 28.7%, anti-TPO positivity in 35.22%, and rheumatoid factor positivity in 9.57%.

**Table 3** shows that no significant association was observed between dermographism and autoimmune status in CSU patients, with similar proportions of autoimmune positivity in patients with and without dermographism (72.4% vs. 65.9%, respectively; χ²=1.1, p=0.29).

**Table 3:** Association between dermographism and autoimmune status in CSU patients.

Variable	Autoimmune status			p-level
	n	Positive n (%)	Negative n (%)	
Dermogaphism				
Present	161	105 (72.41)	56 (65.88)	X^2^ = 1.1 p=0.29
Not present	69	40 (27.59)	29 (34.12)	

### 3.4 Dermographism and ASST positivity in CSU patients

No significant association was observed between dermographism and ASST positivity in CSU patients, with comparable rates of ASST positivity in patients with and without dermographism (72.9% vs. 65.6%, respectively; χ²=1.4, p=0.24), as shown in **Table 4**.

**Table 4:** Dermographism and ASST positivity in CSU patients.

Variable	ASST			p-level
	n	Positive n (%)	Negative n (%)	
Dermographism				
Present	161	102 (72.86)	59 (65.56)	X^2^ = 1.4 p=0.24
Not present	69	38 (27.14)	31 (34.44)	

### 3.5 Disease duration in CSU patients and dermographism

Patients with dermographism had a significantly longer CSU disease duration compared with those without dermographism (58.5 ± 47.1 vs. 42.8 ± 40.9 months; median 54 [IQR 15–91] vs. 24 [IQR 9–72]; Z = 2.3, p=0.022) as shown in **Table 5**.

**Table 5:** Disease duration in CSU patients and dermographism.

Variable	Disease duration (months)			p-level
	n	mean ± SD	median (IQR)	
Dermographisam				
Present	161	58.5 ± 47.1	54 (15 – 91)	Z=2.3 *p=0.022
Not present	69	42.8 ± 40.9	24 (9 – 72)	

## 4 Discussion

The present study demonstrates that dermographism represents a clinically relevant manifestation within the spectrum of CSU and is associated with increased disease activity, prolonged disease duration, and markers of immune dysregulation. Although dermographism has traditionally been classified among inducible urticarias, increasing evidence suggests that dermographic responses may coexist with CSU and contribute substantially to overall disease burden and symptom persistence (1, 3, 4, 8). Our findings support the concept that dermographism identifies a subgroup of CSU patients with enhanced cutaneous mast-cell reactivity and a potentially more severe disease phenotype.

In the current cohort, dermographism was more frequently observed among patients with severe UAS7 scores compared with those exhibiting mild or well-controlled disease activity. This observation aligns with previous investigations reporting that exaggerated dermal hypersensitivity reactions correlate with higher wheal activity and intensified pruritus (19–21). Mechanical stimulation of the skin in dermographic patients likely reflects a lower threshold for mast-cell degranulation, resulting in amplified histamine release and increased vascular permeability. Consequently, dermographism may serve as an indirect clinical marker of heightened mast-cell activation in CSU.

An important observation in this study was the association between dermographism and prolonged CSU disease duration. Patients exhibiting dermographic responses experienced significantly longer CSU disease courses compared with patients without dermographism. Similar findings have been reported in previous longitudinal studies indicating that concomitant physical urticaria features may predict chronicity and delayed remission in CSU (21–23). Persistent mechanical hypersensitivity may therefore indicate ongoing immune activation and sustained inflammatory signaling within the skin microenvironment.

The relationship between dermographism and autoimmune status in CSU also deserves particular attention. Although CSU patients with positive autoimmune markers, including anti-TPO antibodies, ANA positivity, RF positivity, or established autoimmune disease, appeared to show a higher prevalence of dermographism, this difference was not statistically significant. These findings further support the increasingly recognized autoimmune basis of CSU and suggest that dermographism may represent part of a broader autoreactive phenotype (24–26). While autoantibody-mediated mast cell activation and basophil dysfunction have been proposed as potential mechanisms contributing to both spontaneous wheal formation and enhanced responses to physical stimuli, the present findings do not support a significant association between autoimmune status and dermographism. Accordingly, this observation should be interpreted with caution and cannot be regarded as evidence of a relationship between autoimmune status and dermographism.

Although ASST-positive CSU patients numerically exhibited higher rates of dermographism, statistical significance was not consistently reached. This finding mirrors the heterogeneous results reported in previous studies evaluating the relationship between autoreactivity and physical urticaria manifestations (14, 17). The lack of significance may reflect variability in ASST sensitivity, differences in patient selection, or the multifactorial mechanisms underlying dermographic skin responses. Nevertheless, the observed trend suggests that autoreactivity could still contribute to dermographism development in at least a subset of CSU patients.

Interestingly, dermographism did not demonstrate a clear association with therapeutic response to H1-antihistamines in patients with CSU. Comparable frequencies of dermographism were observed among responders and non-responders, suggesting that conventional antihistamine therapy may incompletely suppress mechanically induced mast-cell activation in certain patients. Previous studies have similarly reported that symptomatic dermographism may persist despite otherwise satisfactory CSU control (1, 27, 28). These observations highlight the possibility that mediators beyond histamine—including leukotrienes, neuropeptides, cytokines, and complement-derived factors—may contribute to persistent dermographic activity.

The multiethnic composition of the present cohort represents an additional strength of this study. Patients of Macedonian, Albanian, Turkish, and Roma ethnic backgrounds were included, reflecting the demographic structure of North Macedonia. Importantly, no substantial ethnic differences were identified regarding CSU severity, dermographism prevalence, or autoimmune associations. This finding suggests that the observed clinical correlations are unlikely to be restricted to a single ethnic population and may therefore possess broader regional relevance (4).

Collectively, our findings indicate that dermographism in CSU should not be regarded merely as an incidental physical finding but rather as a clinically meaningful component associated with increased disease burden and prolonged disease persistence. Recognition of dermographism during routine clinical assessment may assist clinicians in identifying patients with potentially more severe or refractory disease phenotypes who could benefit from closer monitoring and earlier therapeutic escalation.

### 4.1 Clinical implications

Given the observed associations between dermographism, increased CSU disease activity, autoimmune status, and prolonged disease duration, patients presenting with symptomatic dermographism may require closer clinical follow-up and earlier therapeutic escalation. In CSU patients with persistent symptoms despite high-dose H1-antihistamines, biologic agents such as omalizumab, dupilumab, remibrutinib or immunomodulatory therapies including cyclosporine may represent appropriate treatment options (1, 3, 27, 28). Routine assessment for autoimmune markers may further contribute to disease stratification and individualized therapeutic planning.

### 4.2 Limitations and future perspectives

Several limitations should be acknowledged.

First, the present investigation was designed primarily to evaluate CSU comprehensively, with dermographism analyzed as one of several associated clinical features rather than as an isolated disease entity. Consequently, the findings reflect the characteristics of the broader CSU population and may not fully capture features unique to patients with symptomatic dermographism alone.

Second, the cross-sectional design limits causal interpretation regarding the relationship between dermographism and disease persistence. Longitudinal multicenter studies are required to determine whether dermographism independently contributes to chronicity or merely reflects a more severe inflammatory phenotype.

Third, the therapeutic analysis was restricted to H1-antihistamine response, without inclusion of second-line and third-line therapies. Therefore, it remains unclear whether persistent dermographism reflects generalized treatment resistance or insufficient control with antihistamines alone.

Additionally, validated instruments specifically designed for inducible urticarias and symptomatic dermographism, including the FricTest, Dermographometer or disease-specific activity scores like Urticaria Control Test (UCT), Symptomatic Dermographism Activity Score (SDAS), Symptomatic Dermographism Quality of Life score (SD-QoL) were not systematically incorporated into the study protocol. Future investigations employing standardized assessment tools may provide more precise characterization of dermographic disease activity and control.

Another limitation of the study is that autoimmune chronic spontaneous urticaria (CSU) endotypes could not be further characterized, as data on total serum IgE levels and IgE autoantibodies against self-antigens were not available, and therefore type I and type IIb autoimmune CSU could not be distinguished, particularly in relation to dermographism.

## 5 Conclusions

This study identified dermographism as a clinically important manifestation associated with increased CSU disease activity, autoimmune background, and prolonged disease duration in patients with CSU. The coexistence of dermographism with CSU appears to reflect enhanced cutaneous autoreactivity and mast-cell hypersensitivity rather than a purely isolated physical urticaria phenomenon. Although dermographism demonstrated limited association with antihistamine responsiveness and ASST positivity in CSU patients, its strong relationship with disease severity and chronicity highlights its importance in routine CSU evaluation and long-term disease monitoring.

Recognition of dermographism may contribute to improved phenotypic stratification and optimization of individualized therapeutic strategies in CSU patients. Further multicenter prospective studies incorporating validated assessment instruments and advanced therapeutic approaches are warranted to better define the prognostic significance and therapeutic implications of dermographism in CSU.

## Data Availability

The datasets generated and analyzed during the current study are not publicly available because they contain information that could compromise participant privacy. De-identified data may be made available by the corresponding author upon reasonable request and subject to approval by the relevant ethics committee and institutional regulations. Requests to access the datasets should be directed to vesna.dojcinovska@gmail.com.
